# Molecular Characterization of a Heterothallic Mating System in *Pseudogymnoascus destructans*, the Fungus Causing White-Nose Syndrome of Bats

**DOI:** 10.1534/g3.114.012641

**Published:** 2014-07-21

**Authors:** Jonathan M. Palmer, Alena Kubatova, Alena Novakova, Andrew M. Minnis, Miroslav Kolarik, Daniel L. Lindner

**Affiliations:** * Center for Forest Mycology Research, Northern Research Station, US Forest Service, Madison, Wisconsin 53726; † Department of Botany, Faculty of Science, Charles University in Prague, Benátská 2, CZ-128 01 Praha 2, Czech Republic; ‡ Institute of Soil Biology, Biology Centre Czech Academy of Sciences, Na Sadkach 7, CZ-370 05 Česke Budějovice, Czech Republic; § Laboratory of Fungal Genetics and Metabolism, Institute of Microbiology of the AS CR, v.v.i, Vídeňská 1083, CZ-142 20 Praha 4, Czech Republic

**Keywords:** geomyces, sexual reproduction, mating type, white-nose syndrome, Genetics of Sex

## Abstract

White-nose syndrome (WNS) of bats has devastated bat populations in eastern North America since its discovery in 2006. WNS, caused by the fungus *Pseudogymnoascus destructans*, has spread quickly in North America and has become one of the most severe wildlife epidemics of our time. While *P. destructans* is spreading rapidly in North America, nothing is known about the sexual capacity of this fungus. To gain insight into the genes involved in sexual reproduction, we characterized the mating-type locus (*MAT*) of two *Pseudogymnoascus* spp. that are closely related to *P. destructans* and homothallic (self-fertile). As with other homothallic Ascomycota, the *MAT* locus of these two species encodes a conserved α-box protein (*MAT1-1-1*) as well as two high-mobility group (HMG) box proteins (*MAT1-1-3* and *MAT1-2-1*). Comparisons with the *MAT* locus of the North American isolate of *P. destructans* (the ex-type isolate) revealed that this isolate of *P. destructans* was missing a clear homolog of the conserved HMG box protein (*MAT1-2-1*). These data prompted the discovery and molecular characterization of a heterothallic mating system in isolates of *P. destructans* from the Czech Republic. Both mating types of *P. destructans* were found to coexist within hibernacula, suggesting the presence of mating populations in Europe. Although populations of *P. destructans* in North America are thought to be clonal and of one mating type, the potential for sexual recombination indicates that continued vigilance is needed regarding introductions of additional isolates of this pathogen.

Since its discovery in 2006 in New York, white-nose syndrome (WNS) of hibernating bats has spread to more than 25 states and 5 Canadian provinces, killing more than 5.5 million bats (Froschauer and Coleman 2012; www.whitenosesyndrome.org). The causative agent of WNS is a psychrophilic fungus named *Pseudogymnoascus destructans* (=*Geomyces destructans*) (Gargas *et al.* 2009; Lorch *et al.* 2011; Warnecke *et al.* 2012; Minnis and Lindner 2013) that has been hypothesized to be an introduced pathogen, possibly from Europe, where the pathogen has been consistently detected (Martínková *et al.* 2010; Puechmaille *et al.* 2010, 2011; Wibbelt *et al.* 2010) and can cause WNS (Pikula *et al.* 2012), although no mass mortality has been observed in Europe. The fungus is spreading rapidly in North America (Lorch *et al.* 2013); isolates collected thus far appear to have been derived from a single clonal introduction in the northeastern United States (Rajkumar *et al.* 2011; Ren *et al.* 2012). This has recently been supported by whole genome sequencing of 26 North American isolates of *P. destructans* (Chibucos *et al.* 2013; K. Drees and J. Foster, unpublished data). Taken together, these data suggest that *P. destructans* is spreading in North America exclusively through asexual reproduction, given that conidia are commonly observed in clinical specimens and in culture (Meteyer *et al.* 2009; Chaturvedi *et al.* 2010). Although it is known that *P. destructans* reproduces asexually, its capacity for sexual reproduction is unknown.

Because fungi and humans are related members of the opisthokonts, sexual reproduction in fungi has been a topic of intense research interest (Heitman *et al.* 2013; Dyer and O’Gorman 2012; Ni *et al.* 2011; Nielsen and Heitman 2007). Although sex involving two mating partners, male and female, is obligatory in some eukaryotes (*e.g.*, humans), mating in fungi can involve multiple mating types, but there are no male and female genders and, thus, no sex chromosomes (*e.g.*, human X & Y). Fungal mating types are determined by a single genetic locus termed the mating-type locus (*MAT* locus), which consists of highly divergent nonhomologous genes that are termed idiomorphs (Heitman *et al.* 2013). Generally, the *MAT* idiomorphs encode for two key transcriptional regulators: where the *MAT1-1* mating type is controlled by the *MAT1-1-1* α-box transcription factor and where the *MAT1-2* mating type is controlled by the *MAT1-2-1* high-mobility group (HMG) transcription factor (Ni *et al.* 2011). Whereas some fungal species have a heterothallic (outcrossing) mating system [*e.g.*, *Neurospora crassa* (Metzenberg and Glass 1990) and *Aspergillus fumigatus* (O’Gorman *et al.* 2009)] involving each individual having either the *MAT1-1-1* or the *MAT1-2-1* idiomorph, others can reproduce homothallically; the individual carries both idiomorphs, and thus a single strain is capable of mating with itself, *i.e.*, it is self-fertile [*e.g.*, *Aspergillus nidulans* (Paoletti *et al.* 2007), *Sclerotinia sclerotiorum* (Amselem *et al.* 2011), *Sordaria macrospora* (Klix *et al.* 2010)]. The specific gene organization of the *MAT* locus can be variable among fungal species, although the canonical *MAT1-1-1* and *MAT1-2-1* are always present (Figure 1). In several species, additional proteins are encoded in the *MAT* locus, for example, *Neurospora crassa* (Ferreira *et al.* 1996) and *Sordaria macrospora* (Klix *et al.* 2010) contain an additional HMG-box gene (*MAT1-1-3*) (Figure 1).

**Figure 1:**
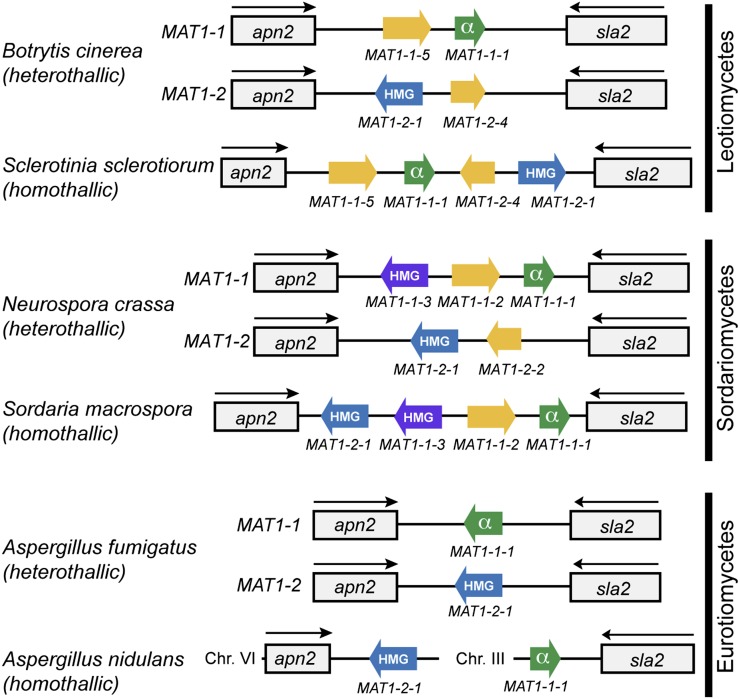
The *MAT* locus of filamentous fungi consists of the conserved regulators *MAT1-1-1* and *MAT1-2-1*. Sex in members of the filamentous Ascomycota can be either homothallic (self-fertile) or heterothallic (require the opposite mating type). The mechanism of sexual reproduction requires the actions of the conserved transcription factors, *MAT1-1-1* and *MAT1-2-1*, which are physically linked to the flanking genes *apn2* and *sla2* of the *MAT* locus. *MAT1-1-1* contains an α-box domain, whereas *MAT1-2-1* contains a related HMG-box domain. Some Ascomycota also have an additional HMG-box transcription factor (*MAT1-1-3*) that has also been implicated in sexual reproduction.

Although the infectious particles of many fungal pathogens are asexual spores, sexual spores can be infectious, as in the human pathogenic fungus *Cryptococcus neoformans* (Giles *et al.* 2009; Velagapudi *et al.* 2009). This is also true of many fungal pathogens of plants, such as *Venturia inaequalis* (apple scab) (Machardy *et al.* 2001) and *S. sclerotiorum* (white mold) (Amselem *et al.* 2011). In many pathogenic fungi, sexual spores also function as important overwintering or survival structures, allowing the fungus to persist for long periods of time in the absence of a host. Moreover, sexual reproduction in pathogenic fungi is of interest because it is the basis for genetic variability that has the potential to create additional virulent genotypes. Mating populations of *P. destructans* in North America could potentially exacerbate WNS, so information regarding the sexual capabilities of this fungus is needed to help inform management and to develop effective mitigation strategies, especially in relation to long-distance (inter-continental) movement of *P. destructans*.

Based on a recent phylogenetic study of the genus *Pseudogymnoascus* (Minnis and Lindner 2013), we selected two unnamed homothallic (self-fertile) species that produce sexual structures (gymnothecia) in culture and are relatively closely related to *P. destructans* as exemplars for understanding the mating-type locus in *Pseudogymnoascus*. We cloned and sequenced the mating-type (*MAT*) locus from these two homothallic species and discovered that these species share a nearly identical gene structure at the *MAT* locus (see *Results* section). Comparison of the homothallic *Pseudogymnoascus MAT* locus to the *P. destructans* genome reference strain suggested that the bat pathogen was likely heterothallic. We screened isolates of *P. destructans* from central Europe and discovered the opposite mating type (*MAT1-2*). Differential expression of *P. destructans* genes involved in mating was also examined in cultures of each mating type individually as well as in mixed culture.

## Materials and Methods

Fungal strains used in this study are listed in Table 1. New strains of *P. destructans* were isolated from muzzles and wings of bats with suspected WNS (*Myotis myotis* and *Plecotus auritus*) using sterile cotton or plastic swabs and cultured on yeast extract glucose chloramphenicol agar or Sabouraud dextrose agar at 10°. For routine laboratory experiments, isolates were maintained on a combination of glucose minimal medium (GMM) (Shimizu and Keller 2001) and Champe’s medium (Champe and El-Zayat 1989). All isolates have been preserved in the culture collection of the Center for Forest Mycology Research (CFMR). The Culture Collection of Fungi (CCF) and the Collection of Microscopic Fungi (CMF) Czech Republic also maintain cultures as indicated by their acronyms in Table 1. All primers are listed in Supporting Information, Table S1. PfuUltra II polymerase (Stratagene) was used for all PCR reactions according to the manufacturer’s recommendations. Standard molecular biology techniques were used as previously described (Sambrook and Russell 2001). BLAST searches were conducted using the draft genome sequence of the North American isolate 20631-21 of *P. destructans* (*Geomyces destructans* Sequencing Project, Broad Institute of Harvard and MIT; http://www.broadinstitute.org/).

**Table 1:** Isolates of *Pseudogymnoascus* used in this study

			Collection Information				Sequence Accession #			
Isolate	Species	MAT Type	Location	Date	Substrate	Citation	ITS	LSU	TEF	MAT
20631-21	*P. destructans*	*MAT1-1*	USA, New York	2008	*M. lucifugus*	(Gargas *et al.* 2009)	EU884921	KF017865	KF017806	KJ938437
WSF 3629	*P*. sp.	Homothallic	USA, Wisconsin	1960	Amorphous peat	(Christensen and Whittingham 1965)	KF039897	KF017870	KF017811	KJ938436
23342-1-I1	*P*. sp.	Homothallic	USA, Wisconsin	2008	*P. subflavus*	(Muller *et al.* 2012)	JX415266	KF017868	KF017809	KJ938435
CCF4801	*P. destructans*	*MAT1-1*	CR, SW Bohemia	2013	*M. myotis*	This study				
CMF2498	*P. destructans*	*MAT1-1*	Slovakia, Harmanecka Cave	2013	*M. myotis*	This study				
CMF2583	*P. destructans*	*MAT1-1*	CR, Moravia, Na Pomezí Caves	2013	*M. myotis*	This study				
CMF2584	*P. destructans*	*MAT1-2*	CR, Moravia, Na Pomezí Caves	2013	*M. myotis*	This study	KJ938418	KJ938423	KJ938428	
CCF3937	*P. destructans*	*MAT1-1*	CR, Bohemian Karst, Mala Amerika	2010	*M. myotis*	(Kubátová *et al.* 2011)				
CCF3938	*P. destructans*	*MAT1-1*	CR, Solenice	2010	*M. myotis*	(Kubátová *et al.* 2011)				
CCF3941	*P. destructans*	*MAT1-1*	CR, Bohemian Karst, Mala Amerika	2010	*M. myotis*	(Kubátová *et al.* 2011)				
CCF3942	*P. destructans*	*MAT1-2*	CR, Bohemian Karst, Mala Amerika	2010	*M. myotis*	(Kubátová *et al.* 2011)	KJ938422	KJ938427	KJ938432	KJ938434
CCF3944	*P. destructans*	*MAT1-1*	CR, Novy Knin	2010	*M. myotis*	(Kubátová *et al.* 2011)				
CCF4103	*P. destructans*	*MAT1-1*	CR, Herlikovice	2011	*P. auritus*	This study				
CCF4124	*P. destructans*	*MAT1-2*	CR, Horni Alberice	2011	*M. myotis*	This study	KJ938421	KJ938426	KJ938431	KJ938433
CCF4125	*P. destructans*	*MAT1-1*	CR, Horni Alberice	2011	*M. myotis*	This study				
CCF4127	*P. destructans*	*MAT1-1*	CR, Herlikovice	2011	*M. myotis*	This study				
CCF4128	*P. destructans*	*MAT1-1*	CR, Herlikovice	2011	*M. myotis*	This study				
CCF4129	*P. destructans*	*MAT1-1*	CR, Pistov	2011	*M. myotis*	This study				
CCF4130	*P. destructans*	*MAT1-1*	CR, Fucna-Otov	2011	*M. myotis*	This study				
CCF4131	*P. destructans*	*MAT1-2*	CR, Vyskov	2011	*M. myotis*	This study	KJ938420	KJ938425	KJ938430	
CCF4132	*P. destructans*	*MAT1-1*	CR, Pernink	2011	*M. myotis*	This study				
CCF4247	*P. destructans*	*MAT1-1*	CR, Morina	2012	*M. myotis*	This study				
CCF4350	*P. destructans*	*MAT1-1*	CR, Bohemian Karst, Mala Amerika	2012	*M. myotis*	This study				
CCF4351	*P. destructans*	*MAT1-2*	CR, Bohemian Karst, Mala Amerika	2012	*M. myotis*	This study	KJ938419	KJ938424	KJ938429	
CCF4471	*P. destructans*	*MAT1-1*	CR, Bohemian Karst, Velka Amerika	2013	*M. myotis*	This study				

ITS, internal transcribed spacer region; LSU, nuclear large subunit region; TEF, translation elongation factor EF-1α; MAT, mating-type locus; CR, Czech Republic.

### DNA extraction from fungi

Fungal cultures were grown in liquid stationary culture for 3 wk in Champe’s medium (Champe and El-Zayat 1989), mycelium was collected, lyophilized overnight, ground to a fine powder, mixed with 700 ul of LETS Buffer (100 mM lithium chloride, 20 mM EDTA, 10 mM Tris-HCL, pH 8.0, and 0.5% SDS), and extracted with an equal volume of phenol:chloroform:isoamyl alcohol (Ambion); the aqueous phase was collected after centrifugation for 10 min at 12,000*g* at 4°. DNA was precipitated by adding 1.0 ml of 95% ethanol and centrifuged for 10 min (12,000*g* at 4°). The DNA pellet was washed with 70% ethanol and subsequently resuspended in 10 mM Tris-HCl (pH 8.0) containing 20 units of RNAseA (5′).

### Cloning of *MAT* locus in homothallic *Pseudogymnoascus* species

Primers designed at conserved internal locations of *P. destructans MAT1-1-1* (α-box) were used to amplify a PCR fragment of the *MAT1-1-1* gene from the homothallic *Pseudogymnoascus* species (WSF 3629 and 23342-1-I1); ∼900-bp fragment was obtained for isolate WSF 3629 and ∼400-bp fragment was obtained for 23342-1-I1. The PCR fragments were subsequently cloned using pCR-Blunt II-TOPO (Life Technologies) and sequenced. Sequencing of the region flanking the *MAT1-1-1* gene was achieved by using a modified version of thermal asymmetrical interlaced PCR (TAIL-PCR) called fusion primer and nested integrated PCR (FPNI-PCR) (Wang *et al.* 2011). Briefly, degenerate fusion primers (FP1–FP9) were pooled in batches of three and used in combination with gene-specific primers (GSP) followed by two consecutive nested PCR reactions. The largest PCR product from the final nested reaction was gel-purified, cloned into pCR-Blunt II-TOPO, and subsequently sequenced. Five successive rounds of FPNI-PCR were conducted for isolate 23342-1-I1 and four rounds were conducted for WSF 3629, which was sufficient to identify the conserved flanking gene *sla2*. The remaining portion of the *MAT* locus for each isolate was PCR-amplified by using a primer anchored in the conserved flanking gene *apn2* and a GSP primer from the FPNI-PCR walking, cloned into pCR-Blunt II-TOPO, and sequenced. Gene prediction was performed using a combination of FGENESH (Solovyev *et al.* 2006) and AUGUSTUS 2.7 (Stanke *et al.* 2004) using the pre-trained hidden-Markov models for *Botrytis cinerea*.

### Identification of *P. destructans MAT1-2* locus

Twenty-three isolates of *P. destructans* from central Europe were screened via Southern blot using a 900-bp PCR fragment of *MAT1-1-1* as a radio-labeled ^32^P probe according to standard procedures (Sambrook and Russell 2001). Isolates missing this fragment were suspected of having the other mating type. The *MAT1-2* locus *of P. destructans* was cloned and sequenced from isolates CCF3942 and CCF4124 by PCR amplifying the entire region between *apn2* and *sla2*. The previous Southern blot was stripped and re-probed with a 1.1-kb radio-labeled ^32^P probe corresponding to the *MAT1-2-1* sequence.

### RNA extraction and semi-quantitative RT-PCR

Conidia were harvested in sterile water supplemented with 0.01% Tween-80 from 8-week-old cultures of *P. destructans* grown on GMM medium at 15°. Conidia from a *MAT1-1* isolate and a *MAT1-2* isolate were enumerated with a hemocytometer and used to inoculate 50-ml liquid cultures of Champe’s medium at a concentration of 1 × 10^5^ conidia per ml. Cultures were incubated in a shaker at 15° and 200 rpm for 14 d. Mycelium was collected from each strain by sterile filtration over Miracloth (CalBiochem) and subsequently transferred to the surface of solid GMM medium agar plates: one plate for each mating type as well as one that was a 1:1 mixture of mycelium from *MAT1-1* and *MAT1-2* strains. The plates were wrapped in Parafilm-M (Bemis) and aluminum foil and incubated at 15° for an additional 14 d. Mycelium was then scrapped off the surface of the plates using a sterile glass slide, immediately frozen in liquid nitrogen, and lyophilized overnight. Total RNA was extracted from the lyophilized tissue using Isol-RNA Lysis Reagent (5 Prime) following manufacturer’s recommendations, treated with DNase I (NEB) according to the manufacturer’s protocol, and subsequently used to make cDNA using the iScript cDNA Synthesis Kit (Biorad). Genes involved in sexual reproduction in other filamentous fungi were identified through BLASTp searching of the *P. destructans* reference genome and primers were designed for the mating-type genes (*MAT1-1-1*–GMDG_01209.1, *MAT1-1-3*–GMDG_01208.1, and *MAT1-2-1*–KJ938434), the pheromone pathway (*ppg1*–GMDG_06142.1, *pre1*–GMDG_00660.1, and *pre2*–GMDG_08410.1), the G-protein signaling pathway (*fad1*–GMDG_04604.1, *sfa4*–GMDG_08182.1, *gpg1*–GMDG_01954.1, *mpk2*–GMDG_04404.1, and *ste1*–GMDG_05416.1), and the velvet complex (*vel1*–GMDG_00043.1, *vel2*–GMDG_08054.1, and *lae1*–GMDG_07817.1); actin (*act1*–GMDG_01001.1) was used as a loading control. Between 32 and 42 amplification cycles were used to detect transcription of genes putatively involved in sexual reproduction.

## Results

### Identification of the mating-type locus

The *MAT* locus of *P. destructans* was identified by a BLASTp (Altschul *et al.* 1997) search of the *P. destructans* draft genome assembly with the *MAT* α-box (*MAT1-1-1*) protein sequence from *Aspergillus nidulans* AN2755 (Paoletti *et al.* 2007). This resulted in identification of a single hit on Supercontig 14, corresponding to GMDG_01209.1. In other filamentous fungi, conserved primary metabolism genes *apn2* and *sla2* flank the *MAT* locus (Figure 1); thus, we looked at flanking genes on Supercontig 14 and identified GMDG_01207.1 as *apn2* and GMDG_01210.1 as *sla2*. Using the Conserved Domain Database (CDD) search (Marchler-Bauer *et al.* 2011) with GMDG_01209.1, we identified the *MAT* α-box domain (pfam04769). Interestingly, GMDG_01208.1 is also located in the *MAT* locus and has a predicted HMG-box domain (cd01389). A BLASTp search using GMDG_01208.1 of the nonredundant protein database (nr) at NCBI revealed the top hits to be *MAT1-2-1* proteins (ACA51904.1, AFY11134.2, AGH03115.1, CBY44653.1). Therefore, we initially thought that *P. destructans* could be homothallic because the *MAT* locus harbored both *MAT1-1-1* (α-box) and *MAT1-2-1* (HMG-box) genes. However, because we have never observed fruiting bodies from *P. destructans* 20631-21 in culture and the *MAT* locus of some fungi contains two HMG-box domain genes, we could not rule out that *P. destructans* 20631-21 was a *MAT1-1* genotype.

### Cloning and sequencing of *Pseudogymnoascus* homothallic *MAT* loci

**Figure 2:**
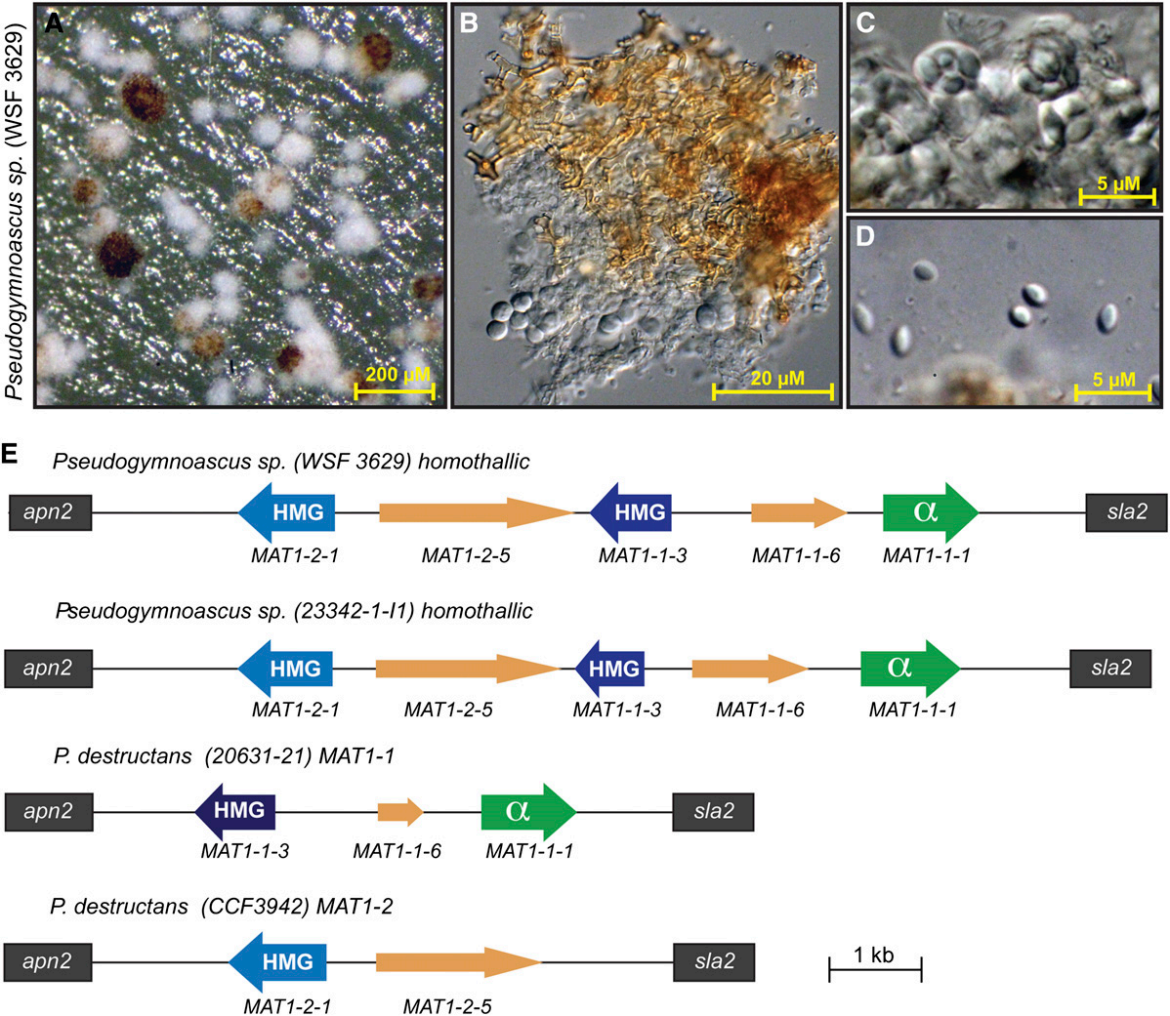
Homothallic species of *Pseudogymnoascus* produced gymnothecia and contain a *MAT* locus consisting of the conserved regulators *MAT1-2-1*, *MAT1-1-3*, and *MAT1-1-1*. (A) Gymnothecia of *Pseudogymnoascus* WSF 3629 grown at 25° for 4 wk on solid oatmeal medium in the dark. Scale bars are drawn on each of the images. (B) Gymnothecia of WSF 3629 are composed of loosely woven, pigmented peridial hyphae, and among the peridial hyphae there are asci. (C and D) Higher magnification of asci containing ascospores and ascospores liberated from asci. (E) Schematic of the mating-type locus (*MAT*) for the homothallic species *Pseudogymnoascus* sp. WSF 3629 and *Pseudogymnoascus* sp. 23342-1-I1. The North American genome reference strain of *P. destructans* (20631-21) is the *MAT1-1* mating type, whereas the *MAT* locus of *MAT1-2* strains is depicted by the Czech strain of *P. destructans* CCF3942.

Several species of *Pseudogymnoascus* are known to be homothallic, and thus produce sexual fruiting bodies in culture (Rice and Currah 2006; Tsuneda 1982). Because homothallic asocmycetes typically have both *MAT* idiomorphs at the *MAT* locus, we reasoned that comparison of the *MAT* locus from a closely related homothallic species would aid in characterization of the *P. destructans* mating system. We selected two unnamed homothallic isolates from a recent study: *Pseudogymnoascus* sp. WSF 3629 (clade G–*P. roseus* complex) and *Pseudogymnoascus* sp. 23342-1-I1 (clade D) (Minnis and Lindner 2013). *Pseudogymnoascus* sp. WSF 3629 does not produce conidia in culture; however, it produces visible gymnothecia (Figure 2A), which are composed of loosely woven, pigmented peridial hyphae (Figure 2B), asci (Figure 2C), and ascospores (Figure 2D). We have observed a similar sexual state for *Pseudogymnoascus* sp. 23342-1-I1; formal identification and/or description of these species are presented elsewhere.

After confirmation of homothallism in two *Pseudogymnoascus* species, PCR primers for the *P. destructans MAT1-1-1* were used to amplify, clone, and sequence a portion of the *MAT1-1-1* gene from both *Pseudogymnoascus* species (WSF 3629 and 23342-1-I1). Subsequent rounds of fusion primer and nested integrated PCR (FPNI-PCR) (Wang *et al.* 2011) were used to obtain sequence of the flanking regions in each direction, yielding the entire sequence between the *apn2* and *sla2* genes from WSF 3629 and 23342-1-I1 (13.2 kB and 12.4 kB, respectively) (Figure 2). Using the *ab-initio* gene prediction programs AUGUSTUS 2.7 (Stanke *et al.* 2004) and FGENESH (www.softberry.com), we deduced that the homothallic *MAT* locus from both WSF 3629 and 23342-1-I1 contain a nearly identical gene structure consisting of five predicted open reading frames (ORFs). A combination of BLAST (Altschul *et al.* 1997), CDD (Marchler-Bauer *et al.* 2011), and InterProScan (Quevillon *et al.* 2005) searches identified a clear *MAT* α-box protein (*MAT1-1-1*) and two high-mobility group (HMG) domain-containing proteins (*MAT1-2-1* and *MAT1-1-3*) (Figure 2). This analysis also identified two additional ORFs (*MAT1-1-6* and *MAT1-2-5*); however, BLAST search did not reveal any significant homology with other known proteins, suggesting that these predicted ORFs are either novel *MAT* genes unique to the Pseudeurotiaceae or perhaps pseudogenes. Pairwise comparison of the *MAT* locus from the homothallic *Pseudogymnoascus* species to the *P. destructans* genome sequenced reference strain (20631-21) indicated that the genome reference strain was missing the *MAT1-2-1* HMG box domain containing gene, as well as the hypothetical *MAT1-2-5* gene, indicating that it was a *MAT1-1* (α-box) mating type.

### Identification of the *P. destructans MAT* idiomorph

**Figure 3:**
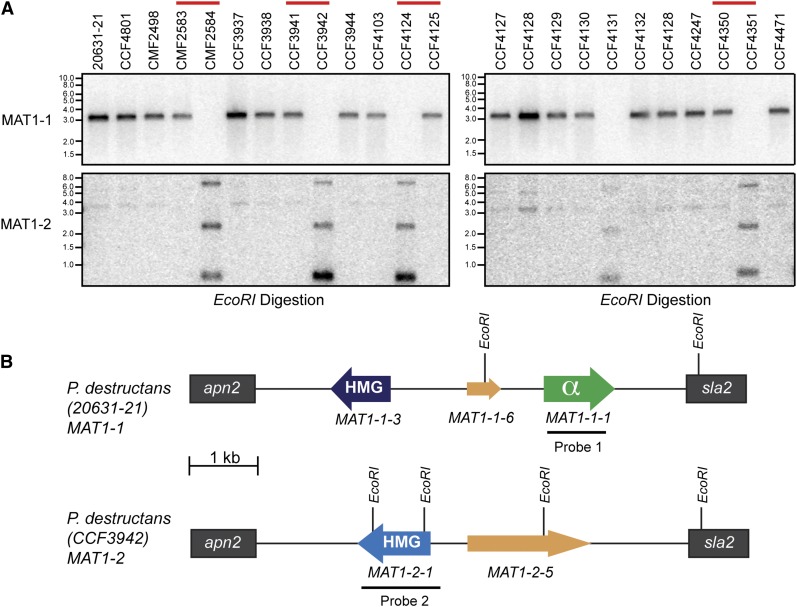
Central European isolates of *P. destructans* have two mating types (*MAT1-1* or *MAT1-2*). (A) Southern blot of the *MAT* locus of the North American isolate (20631-21) and 23 isolates from central Europe. Expected banding patterns for an *EcoRI* digestion of *MAT1-1* strains is a single band of 3.183 kb. Expected banding pattern for *EcoRI* digestion using *MAT1-2* as a probe is three bands of 2.6 kb, 2.063 kb, and 0.699 kb. European isolates of *P. destructans* collected from the same hibernaculum and date, different individual bats, but opposite mating types are demarcated with a red line above the isolate name. (B) Schematic of the two *MAT* idiomorphs in *P. destructans* illustrating the gene prediction structure and restriction enzyme cut sites. Radio-labeled probes used in the Southern blot are indicated by a black line.

The *P. destructans* genome reference strain (20631-21) is a North American isolate that has been hypothesized to be spreading clonally (Ren *et al.* 2012; Rajkumar *et al.* 2011), which has recently been substantiated because analysis of whole genome sequencing data of 26 North American isolates of *P. destructans* revealed that they are all the *MAT1-1* genotype (Chibucos *et al.* 2013; K. Drees and J. Foster, unpublished data). Although diversity studies of *P. destructans* isolates collected from Europe have not been conducted, it has been hypothesized that the fungus may have originated from Europe (Warnecke *et al.* 2012); therefore, we looked for alternative mating types in *P. destructans* isolates from central Europe (Czech Republic and Slovakia). We screened 23 isolates of *P. destructans* for the presence of the *MAT1-1-1* gene via Southern blotting and found that five of the isolates (CMF2584, CCF3942, CCF4124, CCF4131, and CCF4351) were missing *MAT1-1-1* (Figure 3A). These isolates were confirmed to be *P. destructans* by morphology as well as sequencing of the ITS, LSU, and TEF regions (Table 1). We next cloned and sequenced the entire *MAT* locus from CCF3942 as well as the genome reference strain 20631-21 as a control (Table 1). Consistent with its Southern blot, CCF3942 did not contain the *MAT1-1-1* sequence; instead, this isolate harbors a HMG box domain containing protein (*MAT1-2-1*), suggesting that this isolate is the opposite mating type (Figure 3B). Moreover, a Southern blot using a probe for *MAT1-2-1* identified the remaining four isolates as being identical to CCF3942 (Figure 3A). There is also an additional faint band in the Southern blot of *MAT1-1* isolates when probed with *MAT1-2-1*, which could be due to homology in the HMG-box domain of *MAT1-1-3*. It has recently been recognized that the *MAT* transcription factors share an evolutionary history, because even the *MAT1-1-1* α-box is derived from the HMG gene family (Martin *et al.* 2010).

We also cloned and sequenced the *MAT* locus from CCF4124, which was a *MAT1-2* isolate that was collected on a different date and location. These data corroborate that there are two *MAT* idiomorphs for the isolates examined: *MAT1-1* and *MAT1-2*. Interestingly, both mating types were isolated from samples taken at distinct times from different individual bats from the same hibernaculum, even though only 23 isolates of European *P. destructans* were screened in this study (Figure 3A).

### Analysis of genes involved in sexual reproduction

**Figure 4:**
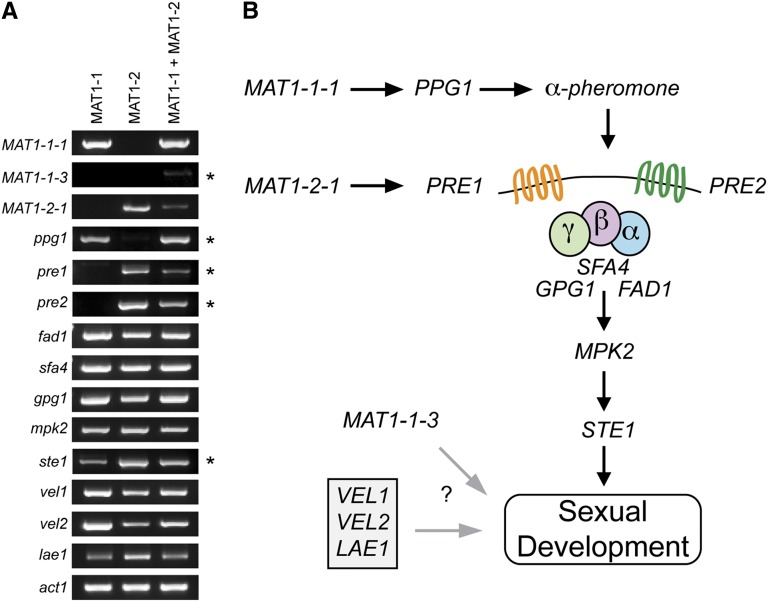
Putative genes involved in sexual reproduction are expressed in laboratory culture. (A) Semi-quantitative reverse-transcriptase PCR was used to measure gene expression of genes predicted to be involved in sexual reproduction. All PCR reactions were conducted with 32 amplification cycles except for those marked with an asterisk (*), where 42 amplification cycles were used. Mating type *MAT1-1* is required for expression of the precursor to alpha-pheromone (*ppg1*), whereas *MAT1-2* is required for expression of the G-protein-coupled receptors *pre1* and *pre2*. Expression of *MAT1-1-3* is only found when both mating types were co-cultured. (B) Proposed diagram of genes involved in sexual reproduction in *P. destructans* based on homology and expression in laboratory culture.

Although this is the first molecular characterization of sexual reproduction in *Pseudogymnoascus*, much is known about the molecular pathways in other model fungal systems such as *Saccharomyces cerevisiae*, *Neurospora crassa*, *Aspergillus nidulans*, and others (Dyer and O’Gorman 2012). Using data from the aforementioned model systems, we sought to examine expression of several conserved genes involved in sexual reproduction by semi-quantitative reverse-transcriptase PCR of *P. destructans* mRNA from two mating-type isolates grown alone or in mixed culture (Figure 4A). These data are consistent with a typical heterothallic mating system in other fungi, where the *MAT1-1* locus controls the expression of the precursor of α-pheromone (*ppg1*), which is involved in production of the α-mating pheromone. The α-pheromone is recognized by the G-protein-coupled receptors (*PRE1* and/or *PRE2*), which our data suggest are, in turn, under the control of the *MAT1-2* locus in *P. destructans* (Figure 4A). In *P. destructans*, it appears that co-cultivation of *MAT1-1* and *MAT1-2* strains results in the weak induction of the *MAT1-1-3* HMG domain–containing gene (Figure 4A). Expression of genes in the signal transduction pathway (*fad1*, *sfa4*, *gpg1*, *mpk2*, and *ste1*) as well as in the velvet complex (*vel1*, *vel2*, and *lae1*) were not drastically altered in either of the mating types or in mixed culture (Figure 4A). Taken together, these data indicate that *P. destructans* has the necessary genetics for sexual reproduction and allow us to propose a heterothallic sexual reproduction pathway (Figure 4B).

## Discussion

To gain insight into the molecular components of sexual reproduction in the Pseudeurotiaceae, we selected two homothallic (self-fertile) isolates from a recent study characterizing species related to *P. destructans* (Minnis and Lindner 2013). Cloning and sequencing of the *MAT* locus in each of these species revealed it was nearly identical and encodes for a conserved α-box domain protein (*MAT1-1-1*) and two conserved HMG box domain proteins (*MAT1-1-3* and *MAT1-2-1*). This is consistent with the *MAT* locus of other well-studied homothallic Ascomycota species such as *Sordaria macrospora* (Klix *et al.* 2010), *Fusarium graminearum* (Yun *et al.* 2000), and *Sclerotinia sclerotiorum* (Amselem *et al.* 2011), where the mating genes are located in one conserved locus flanked by the primary metabolism genes *sla2* and *apn2*. Comparison of the homothallic *MAT* locus with the genome reference strain of *P. destructans* (20631-21) revealed that it was missing the *MAT1-2-1* HMG box protein, suggesting it was heterothallic. Interestingly, there are two more predicted ORFs in the homothallic *MAT* locus, *MAT1-1-6* and *MAT1-2-5*, which appear to have no known functional domains or homology to other known proteins and thus may represent novel *MAT* genes in the Pseudeurotiaceae.

Pertinent to WNS management, we found isolates of both mating types of *P. destructans* coexisting in European hibernacula, indicating that in central Europe there is the potential for mating populations. Although these data suggest that, in our limited sampling, the *MAT1-1* mating type is found more frequently on *Myotis myotis* (18 out of 23), more sampling of European fungal isolates is necessary to understand the prevalence of mating types in *P. destructans*. Preliminary experiments inducing sexual reproduction in the laboratory have not yielded results to date; this is not surprising because *P. destructans* is slow-growing and sexual reproduction may not occur for long time periods, as exemplified by other members of the genus (Rice and Currah 2006). Moreover, finding the appropriate cultural conditions for fungi with cryptic sexual cycles is time-consuming. For example, although the heterothallic *MAT* locus *of Aspergillus fumigatus* was characterized in 2005 (Paoletti *et al.* 2005), it took another 4 yr to find cultural conditions conducive to sexual reproduction (O’Gorman *et al.* 2009). Molecular characterization of the *MAT* locus of isolates will hasten the progress in finding the sexual cycle of *P. destructans*.

In the absence of sexual structures of *P. destructans*, we sought to further investigate genetic pathways involved in sex that have been well-studied in other fungi (Dyer and O’Gorman 2012). Consistent with other Ascomycota, our expression data suggest that *MAT1-1* and *MAT1-2* are likely responsible for determination of mating type, because the precursor to α-pheromone (*ppg1*) was only expressed in the *MAT1-1* background. Moreover, both of the G-coupled-protein receptors (*PRE1* and *PRE2*) hypothesized to recognize the α-pheromone are only expressed in the *MAT1-2* background. Although we did not detect differences in expression of genes involved in sexual development in other fungi, which included the signal transduction cascade (*fad1*, *sfa4*, *gpg1*, *mpk2*, *ste1*) (Dyer and O’Gorman 2012) and the velvet complex of proteins (*lae1*, *vel1*, *vel2*) (Bayram and Braus 2012; Bayram *et al.* 2008), this was not surprising given the central importance of these genes for normal growth of the fungus. Interestingly, *MAT1-1-3*, the HMG-box domain protein of the *MAT1-1* idiomorph, is only expressed at low levels when both mating types are grown in co-culture, suggesting that it could be involved in downstream transcriptional activation of sexual reproduction.

Given the apparent clonality of *P. destructans* in North America, this important discovery of heterothallic mating types highlights the need for continued vigilance in preventing additional introductions of this pathogen in North America. Further work is needed to find and characterize the cryptic sexual cycle of *P. destructans*, although determination of the mating types of isolates will be crucial to successfully characterizing sexual reproduction in this fungal pathogen under laboratory conditions. Sexual recombination may allow *P. destructans* to quickly adapt to its environment and hosts, despite its slow growth. Pertinent to pathogenicity of *P. destructans*, mating types in other fungi have been correlated to virulence (Cheema and Christians 2011; Nielsen *et al.* 2005; Kwon-Chung *et al.* 1992); therefore, this will be an important consideration in elucidating pathogenicity factors of WNS.

## Supplementary Material

Supporting Information
