# Supplementary material for: Molecular Characterization of a Heterothallic Mating System in Pseudogymnoascus destructans, the Fungus Causing White-Nose Syndrome of Bats
Source: G3 (Bethesda). 2014 Jul 21;4(9):1755–63. doi: 10.1534/g3.114.012641 (PMC4169168; doi:10.1534/g3.114.012641)
Supplement: Supporting Information [file supp_4_9_1755__index.html]

Molecular Characterization of a Heterothallic Mating System in Pseudogymnoascus destructans, the Fungus Causing White-Nose Syndrome of Bats — Supporting Information 

# Molecular Characterization of a Heterothallic Mating System in *Pseudogymnoascus destructans*, the Fungus Causing White-Nose Syndrome of Bats

## Supporting Information for Palmer *et al.*, 2014

**Files in this Data Supplement:**

- Table S1 - Primers used in this study. (PDF, 92 KB)
