# Supplementary material for: Molecular Characterization of a Heterothallic Mating System in Pseudogymnoascus destructans, the Fungus Causing White-Nose Syndrome of Bats
Source: G3 (Bethesda). 2014 Jul 21;4(9):1755–63. doi: 10.1534/g3.114.012641 (PMC4169168; doi:10.1534/g3.114.012641)
Supplement: Supporting Information [file supp_g3.114.012641_012641SI.pdf]

G3: Genes | Genomes | Genetics

Molecular characterization of a heterothallic mating system in *Pseudogymnoascus destructans*, the fungus causing white-nose syndrome of bats

Jonathan M. Palmer[*], Alena Kubatova[†], Alena Novakova[‡§], Andrew M. Minnis[*], Miroslav Kolarik[†,§], Daniel L. Lindner[*,1]

[*] Center for Forest Mycology Research, Northern Research Station, US Forest Service, Madison, WI 53726, USA. [†] Department of Botany, Faculty of Science, Charles University in Prague, Benátská 2, CZ - 128 01 Praha 2, Czech Republic. [‡,] Institute of Soil Biology, Biology Centre Czech Academy of Sciences, Na Sadkach 7, CZ - 370 05 Česke Budějovice, Czech Republic. [§] Laboratory of Fungal Genetics and Metabolism, Institute of Microbiology of the AS CR, v.v.i, Vídeňská 1083, CZ - 142 20 Praha 4, Czech Republic.

[1] Corresponding Author
Daniel L. Lindner
One Gifford Pinchot Dr.
Madison, WI 53726-2398
Tel. 608-231-9511
Email. dlindner@wisc.edu

DOI: 10.1534/g3.114.012641

Table S1 Primers used in this study.

Name	Sequence: 5' to 3'	Purpose
JP Gd MAT1-1 400 For	AACAGGATCCGTTCAAAGCG	MAT1-1-1 Int
JP Gd MAT1-1 400 Rev	TGCCATGCCAAGAGGGTCC	MAT1-1-1 Int
JP Gd MAT1-1 900 For	ATCCAGGTCACCCGAATAGG	MAT1-1-1 Int
JP Gd MAT1-1 900 Rev	CCCAGGCTTGCCAGATATTC	MAT1-1-1 Int
JP FP1	GTAATACGACTCACTATAGGGCACGCGTG GTNTCGASTWTSGWGTT	FPNI-PCR
JP FP2	GTAATACGACTCACTATAGGGCACGCGTG GTNGTCGASWGANAWGAA	FPNI-PCR
JP FP3	GTAATACGACTCACTATAGGGCACGCGTG GTWGTGNAGWANCANAGA	FPNI-PCR
JP FP4	GTAATACGACTCACTATAGGGCACGCGTG GTAGWGNAGWANCAWAGG	FPNI-PCR
JP FP5	GTAATACGACTCACTATAGGGCACGCGTG GTNGTAWAASGTNTSCAA	FPNI-PCR
JP FP6	GTAATACGACTCACTATAGGGCACGCGTG GTNGACGASWGANAWGAC	FPNI-PCR
JP FP7	GTAATACGACTCACTATAGGGCACGCGTG GTNGACGASWGANAWGAA	FPNI-PCR
JP FP8	GTAATACGACTCACTATAGGGCACGCGTG GTGTNCGASWCANAWGTT	FPNI-PCR
JP FP9	GTAATACGACTCACTATAGGGCACGCGTG GTNCAGCTWSCTNTSCTT	FPNI-PCR
JP FSP1	GTAATACGACTCACTATAGGGC	FPNI-PCR
JP FSP2	ACTATAGGGCACGCGTGGT	FPNI-PCR
JP 23342-1 MAT GSP1	CCCAGTTCAGCTTGGGAAGG	23342-1 FPNI-PCR
JP 23342-1 MAT GSP2	GGAATGATGCCAATTGCGGG	23342-1 FPNI-PCR
JP 23342-1 MAT GSP3	AACTTGTCCAGCGGAGCCAG	23342-1 FPNI-PCR
JP 23342-1 MAT GSP4	GTGGTTATATGCCAGCTGCC	23342-1 FPNI-PCR
JP 23342-1 MAT GSP5	GATAAGGGCCAGCGCCTTATG	23342-1 FPNI-PCR
JP 23342-1 MAT GSP6	CAGTTCAACCCAACACCAGC	23342-1 FPNI-PCR
JP 23342-1 MAT GSP7	CTAGCGGCAATGGAAGGCTG	23342-1 FPNI-PCR
JP 23342-1 MAT GSP8	CGCCGCAGTACTTGGCAAGG	23342-1 FPNI-PCR
JP 23342-1 MAT GSP9	AGATCGATCGGTTGAAACTCG	23342-1 FPNI-PCR
JP 23342-1 MAT GSP10	GGGAAAGCCAAGGGAAAGCG	23342-1 FPNI-PCR
JP 23342-1 MAT GSP11	ATGCAGAGGGAAGTCGAGGG	23342-1 FPNI-PCR
JP 23342-1 MAT GSP12	ACATTAATCGCGCCTCGCGC	23342-1 FPNI-PCR
JP 23342-1 MAT GSP13	GGAACTCAAGCGCCAGCACG	23342-1 FPNI-PCR
JP 23342-1 MAT GSP14	ATCCCGATTGGAAATGCCAG	23342-1 FPNI-PCR
JP 23342-1 MAT GSP15	GATATCAAGAAGCGTGCAGTC	23342-1 FPNI-PCR
JP 23342-1 MAT Seq 1	CATCGAAATACAAGCGGCAG	23342-1 MAT seq
JP 23342-1 MAT Seq 2	TATATGTGCCAGACCACTCG	23342-1 MAT seq
JP 23342-1 MAT Seq 3	CTTGCCGGTATCCAACAGGG	23342-1 MAT seq
JP 23342-1 MAT Seq 4	CTATAACTGGGTTTCGGCCG	23342-1 MAT seq
JP 23342-1 MAT Seq 5	TTACCGAGCATGATGCAGGC	23342-1 MAT seq
JP 23342-1 MAT Seq 6	ATGTAAGAAGTGACCGCGGG	23342-1 MAT seq
JP 3629 MAT GSP1	ACATTAGTCCAGCCCGGTAG	3629 FPNI-PCR
JP 3629 MAT GSP2	GCCATCCAAGAGTTCAATGG	3629 FPNI-PCR
JP 3629 MAT GSP3	CAATGACATGGTGTGCAGCG	3629 FPNI-PCR
JP 3629 MAT GSP4	TCCGTCATTGATCTGGACCG	3629 FPNI-PCR
JP 3629 MAT GSP5	ACCCACCAGACAACACGTTG	3629 FPNI-PCR
JP 3629 MAT GSP6	GGACAACGAGTACTACAACAC	3629 FPNI-PCR
JP 3629 MAT GSP7	GGAAGTTTAGCCCTTCCTACTCG	3629 FPNI-PCR
JP 3629 MAT GSP8	ATGTTCTATTTGTTGGGGCC	3629 FPNI-PCR
JP 3629 MAT GSP9	CTTATCTCTGTATTCCTGGC	3629 FPNI-PCR
JP 3629 MAT GSP10	TTCTCATCTCGCCCAGCCGC	3629 FPNI-PCR
JP 3629 MAT GSP11	CGAGTTGCAGAATCTCAACC	3629 FPNI-PCR
JP 3629 MAT GSP12	ACAATGTTTTCGCGAAGCGG	3629 FPNI-PCR

JP 3629 MAT Seq	GGATCAGGACACCCAACAGG	3629 MAT seq
JP 3629 MAT Seq 1	CGGTCGTTCTACATATGCGC	3629 MAT seq
JP 3629 MAT Seq 2	AGCCTCATTGTCGTTTGACC	3629 MAT seq
JP 3629 MAT Seq 3	AGAACTTGTCGGTGAGAACG	3629 MAT seq
JP 3629 MAT Seq 4	CACCAACTCAACAACATGGC	3629 MAT seq
JP 3629 MAT Seq 5	CATGCATGGGTATCAAAGCG	3629 MAT seq
JP 3629 MAT Seq 6	CCAACTCAGCACCAATCTCC	3629 MAT seq
JP 3629 MAT Seq 7	GATGAATTGATGAGTAAGCTG	3629 MAT seq
JP 3629 MAT Seq 8	ACCTACGCCACAATGTGGGC	3629 MAT seq
JP 3629 MAT Seq 9	AGAAAGTCTGTCCGATAGCG	3629 MAT seq
JP 3629 MAT Seq 10	AATTTCATTCGTTGGCCGTG	3629 MAT seq
JP apn2 ambig For	CTCCTACCGGAATTTGATAGAAGGCG	3629/23342 MAT
JP apn2 Int For	GATCTACTTCCCACATCCGC	Pd MAT locus
JP sla2 Int Rev	CTGCAACTCGAGAATAATTTGGC	Pd MAT locus
JP Pd MAT Seq 1	ACAGTGGCGTTGTGGAACG	MAT1-1/1-2 seq
JP Pd MAT Seq 2	GGCTCTTCTGGGATCGAAAG	MAT1-1/1-2 seq
JP Pd MAT Seq 3	GTCGTCGGCTACCAATAAGC	MAT1-1 seq
JP Pd MAT Seq 4	TATTTCCAGCCACAACCCTC	MAT1-1 seq
JP Pd MAT Seq 5	CATATTGTTGTCCTGTCCGG	MAT1-1 seq
JP Pd MAT Seq 6	TGCAATGATCTCACTCTCGC	MAT1-1 seq
JP Pd MAT Seq 7	GGCGTGTGTGTGCTGAATG	MAT1-1/1-2 seq
JP Pd MAT2 Seq	AATTCCCGAGTGCACTACAC	MAT1-2 seq
JP Pd MAT2 Seq 1	GGTATAAAACCAACGGTGGG	MAT1-2 seq
JP Pd MAT2 Seq 2	CAGCGGTAATAGAATTCCAGG	MAT1-2 seq
JP Pd MAT2 Seq 3	AAGGCGGAATATCGACCTGC	MAT1-2 seq
JP Pd MAT2 Seq 4	CTGAAATGGATTGGGCCAGG	MAT1-2 seq
JP Pd MAT1-1-1 Int For	AACAGGATCCGTTCAAAGCG	MAT1-1-1 RT PCR
JP Pd MAT1-1-1 Int Rev	TGCCATGCCAAGAGGGTCC	MAT1-1-1 RT PCR
JP Pd MAT1-1-3 Int For	GCTTCAACACCTTCAACACC	MAT1-1-3 RT PCR
JP Pd MAT1-1-3 Int Rev	GGATGTTCGAATATCCAGGG	MAT1-1-3 RT PCR
JP Pd MAT1-2-1 Int For	TCTCAGCTATAGTTTGCACCAA	MAT1-2-1 RT PCR
JP Pd MAT1-2-1 Int Rev	CATGCATGGGTATCAAAGCG	MAT1-2-1 RT PCR
JP Pd actA Int For	AGTAGCAGCCCTCGTCATTG	Actin RT PCR
JP Pd actA Int Rev	GACAACACCGTGCTCGATTG	Actin RT PCR
JP Pd laeA Int For	CGATTACGAAGAGAATGGGC	laeA RT PCR
JP Pd laeA Int Rev	CTAAATAGCCGAATTGGGGC	laeA RT PCR
JP Pd velB Int For	CGGAGATCATCCACCGAGCT	velB RT PCR
JP Pd velB Int Rev	GCAACCGGATGGACAGCATC	velB RT PCR
JP Pd veA Int For	GACTCGATACCCATCGCGTC	veA RT PCR
JP Pd veA Int Rev	CCTTGGTCTCCTCGTACAGG	veA RT PCR
JP Pd steA Int For	ACCACGACTTCACCACCGAC	steA RT PCR
JP Pd steA Int Rev	GCATGATGCGACACAACAGG	steA RT PCR
JP Pd mpkB Int For	AGGGCGCATACGGTGTTGTC	mpkB RT PCR
JP Pd mpkB Int Rev	GGACATCGAGGATGAGTGTC	mpkB RT PCR
JP Pd gpgA Int For	CACCCGCATCGTCATCGTTC	gpgA RT PCR
JP Pd gpgA Int Rev	GCAGCTAAATTCATGACGCC	gpgA RT PCR
JP Pd sfaD Int For	CGGATCACTTAGGAGATGTC	sfaD RT PCR
JP Pd sfaD Int Rev	AGGGAGACAGATGAATCTCG	sfaD RT PCR
JP Pd fadA Int For	TGATGCAGCGCAATGAGATC	fadA RT PCR
JP Pd fadA Int Rev	CGTGGTCTTAACACGAGACC	fadA RT PCR
JP Pd preB Int For	TATCGAAGGAATCCTCGGCG	preB RT PCR
JP Pd preB Int Rev	ATCCACTCATGCGGTCCATC	preB RT PCR
JP Pd preA Int For	ATCTGGCCCCTCATCTTCAC	preA RT PCR
JP Pd preA Int Rev	CGGATATATCTTCCAGCGCC	preA RT PCR
JP Pd ppgA Int For	CCTTCTTTGACAAGCTCGCC	ppgA RT PCR
JP Pd ppgA Int Rev	GGAAGATCAAGCTTCAACGG	ppgA RT PCR
